# Transmission electron microscopy of pili annulati^⋆^

**DOI:** 10.1016/j.abd.2021.10.011

**Published:** 2022-09-10

**Authors:** Joice Brião Göebel Pinto, Hiram Larangeira de Almeida, Antônia Larangeira de Almeida, Pedro de Oliveira Firpo

**Affiliations:** a Post Graduation Program in Health Sciences, Universidade Católica de Pelotas, Pelotas, RS, Brazil; b Department of Dermatology, Universidade Federal de Pelotas, Pelotas, RS, Brazil; c Dermatology League, Universidade Federal de Pelotas, Pelotas, RS, Brazil; d Universidade Católica de Pelotas, Pelotas, RS, Brazil

**Keywords:** Scanning electron microscopy, Transmission electron microscopy, Pili annulati

## Abstract

**Background:**

Little is known about the ultrastructure of pili annulati.

**Objectives:**

To examine with transmission electron microscopy affected hairs of a family, whose diagnosis had been confirmed in five individuals with scanning electron microscopy, which showed surface undulations with “curtain-like” folding of the hair cuticula and to compare the findings with normal control.

**Methods:**

Hairs of two affected patients and one control were embedded in resin and cut lengthwise to produce ultra-thin sections.

**Results:**

The normal hair showed a parallel arrangement of dark lines associated with less electron-dense wide bands. Small cavities could be observed, mostly in the dark lines, affected hairs had a large number of cavities, associated or not with the insertion of melanosomes and loss of parallelism of the dark lines. Higher magnification showed a significant loss of this parallelism, resembling “wood grooves”. Widened dark lines were observed in some areas.

**Study limitations:**

Only a few hairs were examined.

**Conclusions:**

The present results suggest that the microcanaliculi of the hair surface, easily found with scanning electron microscopy, may be secondary not only to the cavities seen in the sections but also to the disorder of proteins that form this region, demonstrated by the changes of the cortex dark lines.

## Introduction

Pili annulati (PA), a misnomer since rings are not seen, is an uncommon hair shaft disorder. It is characterized by alternating light and dark bands along the hair shaft, which appears shiny and speckled, and is most commonly transmitted as an autosomal dominant disease. This appearance may result from air-filled cavities within the hair shaft’s cortex^1^, ^2^ revealed in Transmission Electron Microscopy (TEM).^3^, ^4^

Scanning Electron Microscopy (SEM) shows surface undulations with ‘curtain–like’ folding of the hair cuticula. 3, 5 An association with alopecia areata, autoimmune thyroid disorders, as well as primary immunoglobulin A deficiency has been reported, but a true pathogenic association with PA has not been established.^6^, ^7^ Some cases revealed hair fragility, mainly related to external damage to the previously affected hair.^8^

**Figure 1:**
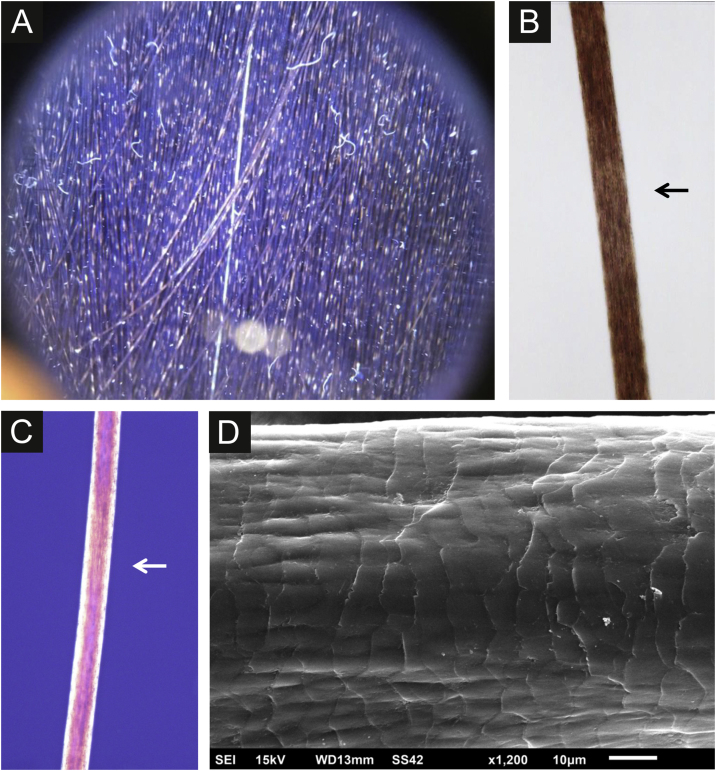
(a) Clinical aspect on trichoscopy. (b) Light microscopy with discrete lighter area (arrow) (×150). (c) Microscopy with polarized light showing area with birefringence (arrow). (d) Scanning electron microscopy ‒ “curtain-like” folding of the cuticula (×1,200).

The authors examined hair specimens from five affected individuals of one family presented with characteristic alternating light and dark bands (Fig. 1a). It was first reported by the hairdressers while the individuals were having a haircut. The inheritance pattern was autosomal dominant, and the patients had no associated comorbidities or hair fragility. Lighter and darker areas were seen in the hair shafts in light microscopy (Fig. 1b). The examination with polarized light showed areas with birefringence (Fig. 1c). The diagnosis was confirmed in all five patients with scanning electron microscopy, which showed surface undulations with “curtain-like” folding of the hair cuticula (Fig. 1d).

## Methods

The hairs of two affected patients and one control were embedded in resin and cut lengthwise to produce ultra-thin sections of the hair shaft cortex to be examined with TEM.

## Results

**Figure 2:**
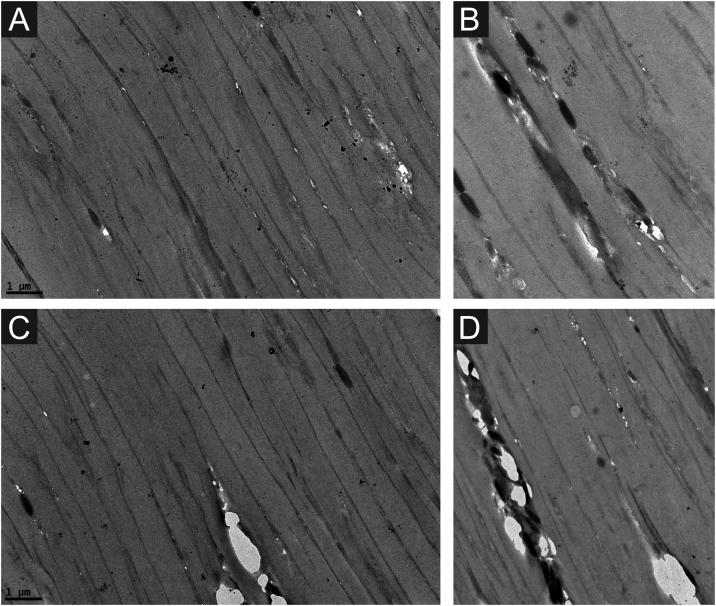
Transmission Electron Microscopy ‒ normal control with a parallel arrangement of dark lines with less electron-dense wide bands (a and c). Small cavities could be observed, mainly in the dark lines (b‒d). These cavities occur spontaneously (c and d), or in areas where the melanosomes are inserted (b and d) (×15,000).

Hair specimens of the unaffected control showed a parallel arrangement of dark lines associated with less electron-dense wide bands (Fig. 2a and c). Small cavities could be observed, mostly in the dark lines (Fig. 2b‒d). These cavities occur spontaneously (Fig. 2c and d) or in areas where the melanosomes are inserted, which are usually seen in the dark lines (Fig. 2b and d).

**Figure 3:**
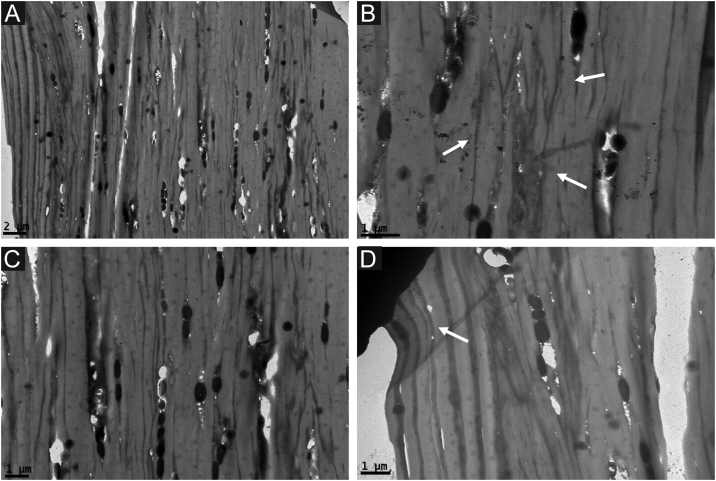
Transmission Electron Microscopy from the first case ‒ Lower magnification with a large number of cavities, associated or not with the insertion of melanosomes (a ) and loss of parallelism of the dark lines ( white arrows,b) (×8,000 and ×15,000). Detail of loss of parallelism of the dark lines, resembling “wood grooves” and cavities( c). Indentation in the cuticle (arrow, d) (×15,000).

Lower magnification revealed that the first affected patient had a large number of cavities, associated or not with the insertion of melanosomes (Fig. 3a and c) and loss of parallelism of the dark lines. Higher magnification showed a significant loss of this parallelism, resembling “wood grooves” (Fig. 3b and c). Indentations were seen in the cuticle near the surface (Fig. 3d).

**Figure 4:**
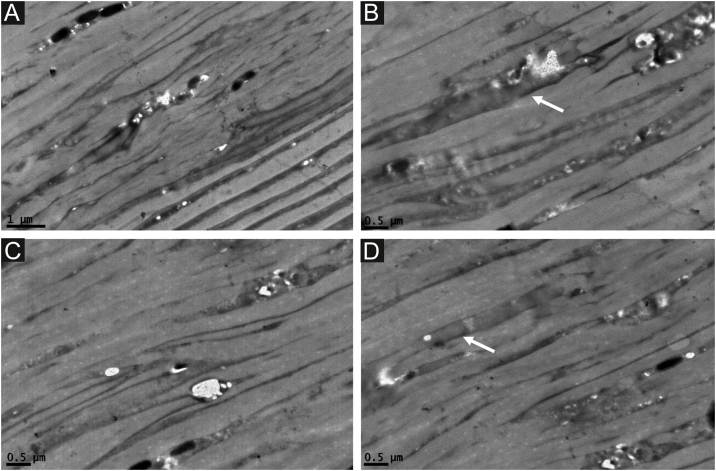
Transmission Electron Microscopy ‒ significant loss of dark line parallelism (a -c), also resembling “wood grooves”, with small cavities not associated with the melanosomes’ insertion ( ×25,000). Widened dark lines (arrows) and cavities (b and d) (×25,000).

The second affected patient also demonstrated a significant loss of dark line parallelism (Fig. 4a-d), also resembling “wood grooves”, with small cavities not associated with the melanosome insertion (Fig. 4a‒c). Widened dark lines were observed in some areas (Fig. 4b and d).

## Discussion

Hair shafts are difficult-to-process tissues for electron microscopy due to frequent breakages and image artifacts,^9^ the reason why there is limited information about TEM in hair diseases.

The authors obtained hair shafts from one unaffected control showing parallelism of the light wide bands and dark narrow bands in the regular cortex. This finding has been previously described as lines following “the hair shaft’s axis”.^10^ Some cavities are found in normal hairs cortex.

The air-filled cavities along the hair cortex are well known in TEM analysis of affected PA.^6^, ^7^, ^8^ These cavities were found in greater number when compared to unaffected hair, and appear isolated or associated with the insertion of melanosomes. However, another important finding in affected hairs was the irregular arrangement of the cortex dark lines, resembling “wood grooves”, these lines were sometimes widened.

Indentations of undamaged cuticles adjacent to cavities have been previously described using electron microscopy,^6^, ^8^ however, most of the studies used cross-sections of the inner structure of the hair shaft,^8^ making it difficult to compare the present findings with longitudinal sections.

SEM shows an intermittent pattern of longitudinal undulations with variable cuticular damage mentioned by some authors as a “cobblestoned”^4^, ^6^ or a “curtain-like” appearance.^5^ These undulations probably correspond to the regions underlying the cortical changes.^8^

The present results suggest that the microcanaliculi of the hair shaft surface, easily found with SEM, may be secondary not only to the cavities seen in the sections but also to the disorder of proteins that form this region, shown by the changes in the cortex's dark lines, which lost their normal parallelism and appeared widened.

## Financial support

None declared.

## Authors' contributions

Joice Brião Göebel Pinto: Approval of the final version of the manuscript; critical literature review; data collection, analysis, and interpretation; intellectual participation in propaedeutic and/or therapeutic management of studied cases; manuscript critical review; preparation and writing of the manuscript; study conception and planning.

Hiram Larangeira de Almeida Jr.: Approval of the final version of the manuscript; critical literature review; data collection, analysis, and interpretation; effective participation in research orientation; intellectual participation in propaedeutic and/or therapeutic management of studied cases; manuscript critical review; preparation and writing of the manuscript; study conception and planning.

Antonia Larangeira de Almeida: Approval of the final version of the manuscript; critical literature review; data collection, analysis, and interpretation; effective participation in research orientation; intellectual participation in propaedeutic and/or therapeutic management of studied cases; manuscript critical review; preparation and writing of the manuscript.

Pedro de Oliveira Firpo: Approval of the final version of the manuscript; critical literature review; data collection, analysis, and interpretation; effective participation in research orientation; intellectual participation in propaedeutic and/or therapeutic management of studied cases; manuscript critical review; preparation and writing of the manuscript.

## Conflict of interest

None declared.
